# Quantitative Analysis of Pyrazines and Their Perceptual Interactions in Soy Sauce Aroma Type Baijiu

**DOI:** 10.3390/foods10020441

**Published:** 2021-02-17

**Authors:** Yan Yan, Shuang Chen, Yao Nie, Yan Xu

**Affiliations:** 1 State Key Laboratory of Food Science & Technology, Key Laboratory of Industrial Biotechnology of Ministry of Education & School of Biotechnology, Jiangnan University, Wuxi 214100, China; 7160201050@vip.jiangnan.edu.cn (Y.Y.); shuangchen@jiangnan.edu.cn (S.C.); 2 School of Liquor and Food Engineering, Guizhou University, Guiyang 550025, China

**Keywords:** pyrazines, UPLC–MS/MS, sub-threshold, perceptive interactions, soy sauce aroma type Baijiu

## Abstract

Pyrazines are important compounds in soy sauce aroma type Baijiu (SSAB). In this work, a total of 16 pyrazines were analyzed using ultra-performance liquid chromatography coupled with tandem mass spectrometry (UPLC–MS/MS) in SSAB. The quantitative results showed that 2,3,5,6-tetramethylpyrazine, 2,6-dimethylpyrazine and 2,3,5-trimethylpyrazine were the three most concentrated pyrazines. The highest odor activity value (OAV) was determined for 2-ethyl-3,5-dimethylpyrazine. Quantitative analysis combined with descriptive sensory analysis revealed that sub-threshold pyrazines (2,3-dimethylpyrazine, 2,3-diethylpyrazine, 2,3-diethyl-5-methylpyrazine and 2-acetyl-3-methylpyrazine) are significantly correlated with the roasted aroma in SSAB. Our study focused on the impact of sub-threshold pyrazines on the perception of roasted aroma in SSAB. The effect of the sub-threshold pyrazines was detected by the addition of various pyrazines in SSAB samples, despite their sub-threshold concentrations. Furthermore, the presence of sub-threshold pyrazines in dilute alcohol solution resulted in a significant reduction in the odor thresholds of supra-threshold pyrazines. Sensory investigation indicated that pyrazines have a synergistic effect on the perception of roasted aroma. The results highlighted the contribution of some pyrazines to the roasted aroma in SSAB despite their sub-threshold concentrations.

## 1. Introduction

Baijiu, also known as Chinese liquor, is a transparent fermented alcoholic beverage dating back thousands of years [1]. Baijiu has a complicated manufacturing process that results in different types of aromas. Soy sauce aroma type Baijiu (SSAB) is a flagship variety that is relished by consumers. The SSAB are mainly characterized by sauce-like, alcoholic, roasted, nutty, floral and fruity aromas [2,3].

The mixture of volatile compounds in SSAB is quite complex. A total of 528 volatile compounds have been identified in SSAB by comprehensive two-dimensional gas chromatography–time-of-flight mass spectrometry [4]. Gas chromatography combined with an olfactory detector (GC-O), which involves the use of GC for separation and the olfaction of trained judges as a detector, is a useful tool for recognizing aroma-active compounds in Baijiu. A total of 186 aroma-active compounds were identified in SSAB by GC-O [5]. Odor activity values (OAVs, defined as the ratio of concentration to odor threshold) are usually determined after GC-O analysis [6]. The sensory effects of compounds with an OAV ≥1 will be further investigated by means of recombination and omission experiments [7]. Sub-threshold refers to concentrations below the olfactory threshold, and concentrations exceeding the olfactory threshold are supra-threshold. Volatile compounds with sub-threshold concentrations are usually known to have little or no impact on total sensory perception [8].

The aroma compounds of many foods are complex, and it is recognized that the aroma is not simply equal to the sum of its aroma compounds [9,10]. Perceptual interactions are indeed found for the aroma compounds. Some methods mainly determined the synergy between aroma compounds through the changes of odor threshold or aroma intensity before and after the combination of aroma compounds. For example, the presence of ethyl-3-hydroxybutanoate and 2-methylpropyl acetate in a wine model solution led to a significant decrease in the olfactory threshold of the fruity aroma [11]. Some studies have shown that certain compounds can play a role in the overall aroma at sub-threshold concentrations. Acetoin, acetic acid and γ-butyrolactone affect the fruity aroma intensity in wine at concentrations of approximately 2%, 12% and 40% of their odor thresholds, respectively [12]. At sub-threshold concentrations, ethylphenol has been shown to suppress the fruity aroma intensity in wine [13]. However, the impact of aroma compounds present at sub-threshold concentrations on odor perception has not previously been investigated in Baijiu.

Pyrazines are six-membered heterocyclic compounds with two nitrogen atoms at positions 1 and 4 [14]. Pyrazines are responsible for universally desirable and enjoyable aromas and have been identified in many foods and beverages, particularly as significant volatile components of various nuts, such as peanuts [15], almonds [16], hazelnuts [17] and pecans [18]. While the significance of a variety of pyrazines affecting roasted and nutty aromas has already been highlighted, existing studies have not explored potential perceptual interactions.

Pyrazines are one of the important classes of compounds in Baijiu and are found in highly variable concentrations, between ng·L^−1^ and mg·L^−1^ [19]. Aroma compounds with similar structures and/or aroma properties may create perceptual interactions in combination [20,21,22]. We were interested in whether the perceptual interaction between pyrazines occurred in Baijiu. Therefore, the aims of this study were (i) to quantify the pyrazines in SSAB by direct injection combined with UPLC–MS/MS, (ii) to estimate the relationships between the pyrazines and roasted aroma by chemosensory analysis, and (iii) to further investigate the possible perceptual interactions between pyrazines.

## 2. Materials and Methods

### 2.1. Chemical and Materials

Pyrazine, 2-methylpyrazine (2M), 2,6-dimethylpyrazine (26DM), 2,3-dimethylpyrazine (23DM), 2-ethyl-3-methylpyrazine (2E3M), 2,3,5-trimethylpyrazine (235TM), 2,3-diethylpyrazine (23DE), 5-ethyl-2,3-dimethylpyrazine (5E23DM), 2,3,5,6-tetramethylpyrazine (2356TTM), 2-isobutyl-3-methylpyrazine (2I3M), 2,3-diethyl-5-methylpyrazine (23DE5M), 2-acetyl-3-methylpyrazine (2A3M), (3,5,6-trimethylpyrazin-2-yl)methanol (TM2YM) and 2-propylpyrazine were purchased from company Sigma-Aldrich (Shanghai, China). Meanwhile, 2-ethyl-6-methylpyrazine (2E6M), 2,6-diethylpyrazine (26DE) and 2-ethyl-3,5-dimethylpyrazine (2E35DM) were purchased from J&K Chemical Corp (Beijing, China). The above commercial standards (≥96% purity) were used. HPLC grade ethanol and LC–MS grade acetonitrile were purchased from Merck (Darmstadt, Germany). Ultra-pure water was obtained from a Milli-Q Gradient A10 system (Millipore, Billerica, MA, USA).

### 2.2. Baijiu Samples

A total of 11 commercial SSAB samples were selected for this study and labeled as GT, DYT, ZJ, XJ, QHZ, ZJ, WZJ, ZZY, WL, LJ and FM. The detailed information is given in Table S1. All Baijiu samples were compliant with the national soy sauce Baijiu standard (GB/T 267602011). After the Baijiu sample was passed through a nylon membrane (13 mm, 0.22 μm; ANPEL, Shanghai, China), internal standard (2-propylpyrazine, 5 mg·L^−1^ in ethanol) was added. All Baijiu samples were performed in triplicate.

### 2.3. Quantification of Pyrazines

Pyrazines were quantitated by UPLC–MS/MS according to the reference, with slight modifications [19]. UPLC was performed by an ACQUITY UPLC system (binary solvent manager; Waters, Milford, CT, USA) coupled to a triple quadrupole mass spectrometer (Xevo TQ-S; Waters, Milford, CT, USA). The solution (injection volume of 10 μL each time) was fractionated using a BEH C18 (100 × 2.1 mm, 1.7 μm; Waters, Milford, CT, USA) column. Electrospray ionization (ESI) generated the ions in a positive mode. The ion source parameters were operated with the previously described method [19]. Separately, mass spectrometer parameters for each pyrazine were optimized by direct infusion of individual standard solutions at concentrations of 200 μg·L^−1^ in acetonitrile. The precursor ion, quantification ion and confirmation ion, cone voltage and collision energy were established. Each pyrazine has two MRM (multiple reaction monitoring) transitions: the most intense transition for quantifications and the second most intense for confirmation purposes. The column temperature was maintained at 40 °C. Mobile phase A consisted of 0.1% formic acid in water and mobile phase B was 0.1% formic acid in acetonitrile. The eluting pump was programmed as follows (0.3 mL·min^−1^): 0–8 min, 3% B; 8–25 min, 3–12% B; 25–31 min, 12–20% B; 31–35 min, 20–70% B; 35–35.5 min, 70–3% B; 35.5–40 min, 3% B.

Three replicates were analyzed for each pyrazine to calculate the relative standard deviation (RSD). The limits of detection (LOD) and limits of quantification (LOQ) of pyrazines were estimated with pyrazine standard solutions when the signal-to-noise ratio reached 3 and 10, respectively. The recovery was calculated: (measured concentration after addition − measured concentration in no spiked Baijiu sample)/added concentration × 100%.

### 2.4. Sensory Analyses

#### 2.4.1. Sensory Panels

All panelists came from Jiangnan University, China. Panel 1 featured 20 judges, 10 males and 10 females, with an average age of 25 years. Panel 2 composed of 12 judges, 6 men and 6 women, aged 27 on average. All staff had rich experience in sensory evaluation, including determination of odor thresholds and quantitative descriptive analysis [23,24]. For sensory training, 2,3,5-trimethylpyrazine (8 mg·L^−1^) in 53% *v*/*v* aqueous ethanol was used as the roasted aroma reference [24]. On the basis of the references, with slight modifications, the panelists scored the intensity of the roasted aroma on a 7-point scale (Table S2) [24,25]. Panel 2 spent ten sessions (1 h each) practicing on how to score the SSAB samples, using the references as anchor points. Panel 2’s performance was assessed by PanelCheck for agreement, discrimination and repeatability among assessors according to the Tucker-1, F- and MSE plots, respectively [26,27], and the results are shown in Figure S1.

#### 2.4.2. Determination of Odor Thresholds and Calculation of OAVs

Some pyrazines’ odor thresholds were detected by a 3-AFC (3-alternative forced-choice) test at seven concentration steps. One glass of the pyrazine and two glasses of 53% ethanol–water solution (*v*/*v*) were prepared. Panel 1 was asked to select the differing one [7]. By dividing the concentration by the value of the pyrazine odor threshold, OAV was established.

#### 2.4.3. SSAB Sample Evaluation

The SSAB samples were smelled by panel 2 with three or four samples per session and the samples were sent in triplicate. The samples were served (15 mL each) in regular Baijiu tasting glasses. Using a 7-point scale, the roasted aroma intensity was graded, varying from 0 (not perceived) to 7 (highest intensity) in contrast to the reference intensity (Table S2).

#### 2.4.4. Discriminative Testing Methods

For various pyrazine addition experiments, 3-alternative forced-choice (3-AFC) studies were conducted by panel 1. In the first series of 3-AFC tests, each pyrazine applied to a SSAB sample (FM) was tested (Last table; tests 1 to 8). In the second step, multiple pyrazine addition experiments were conducted (Last table; tests 9 to 10). Each 3-AFC test provided three samples in random order, two of which were unaltered SSAB samples (FM), with the third being an SSAB sample (FM) with the corresponding pyrazines. Each panelist used direct smell to identify the different one among the three samples.

#### 2.4.5. Determination of the Perceptual Interactions between Pyrazines

The odor threshold for a mixture of four stated supra-threshold pyrazines (2,6-dimethylpyrazine, 2-ethyl-6-methylpyrazine, 2,3,5-trimethylpyrazine and 2-ethyl-3,5-dimethylpyrazine) was established. Odor threshold was calculated in different matrices: dilute alcohol solution and dilute alcohol solution containing four sub-threshold pyrazine mixtures. Odor threshold was determined based on the 3-AFC test using panel 1. Each test included one positive sample, supplemented with volumes of the four supra-threshold pyrazines from 0.039 to 9.824 mL, increasing in each test by 2 times the volume (Table 1). The odor threshold was identified as the compound concentration at which the correct sample could be chosen by 50% of the panelists. Detailed procedures can be found in previous studies [13].

In addition, Feller’s additive model was used to analyze the interaction of the pyrazines after mixing [20,28]. The mixed solution of four sub-threshold pyrazines (2,3-dimethylpyrazine, 2,3-diethylpyrazine, 2,3-diethyl-5-methylpyrazine and 2-acetyl-3-methylpyrazine) was used as A. The mixed solution of four supra-threshold pyrazines (2,6-dimethylpyrazine, 2-ethyl-6-methylpyrazine, 2,3,5-trimethylpyrazine and 2-ethyl-3,5-dimethylpyrazine) using an ascending volume was used as B. For each trial, all panels received a set of three bottles (two blanks containing 53% ethanol/water solution and one supplemented with the pyrazines). The detection probability *p* was determined using 3-AFC. The detection probability of the mixture can be calculated as follows: *p*(AB) = *p*(A) + *p*(B) − *p*(A)p(B), where *p*(AB) indicates the probability of detecting the mixture, and *p*(A) and *p*(B) indicate the probability of detecting components A and B, respectively. If the actual detection performance is below the calculated *p*(AB), a synergistic effect of the two compounds has occurred; if the actual performance matches the calculated *p*(AB), no aroma interaction has occurred; and if the actual performance is above the calculated *p*(AB), some degree of suppression has occurred.

### 2.5. Statistical Analysis

Sensory analysis was submitted to variance analysis (ANOVA). The relationships between pyrazine concentrations and roasted aroma intensity were assessed by Spearman correlation coefficients (ρ) and significance thresholds (*p*) using SPSS Statistics 25 (IBM, Armonk, NY, USA). *p* < 0.05 and ρ > 0.5 were regarded as a positive relationship [24,29]. For the data processing of the Feller’s additive model, the Sigma Plot 12.0 software (Systat, San Jose, CA, USA) was used.

## 3. Results

### 3.1. Quantitation of Pyrazines by UPLC–MS/MS Approach

The most commonly used analytical methods for pyrazines utilize GC. However, since Baijiu is a liquid obtained by distillation, these pyrazines are more suitable for LC–MS analysis [19]. To the best of our knowledge, some pyrazines have not been reported for quantitative analysis by LC–MS; therefore, a vital step in reaching the optimum sensitivity for some pyrazines was to optimize the MS parameters. For example, for 2,3-diethyl-5-methylpyrazine, this was the first time that LC–MS was used for quantitative analysis. To acquire the precursor and quantifier ions with the highest fragment ion abundance, the cone voltage and collision energy were optimized. Two transitions were selected: the most intense transition (*m*/*z* 150.8–136.0) for quantification and the second most intense (*m*/*z* 150.8–122.5) for confirmation purposes (Figure S2). Then, under the same chromatographic conditions, qualitative and quantitative analyses of 2,3-diethyl-5-methylpyrazine were performed by comparing the peak in the SSAB sample with authentic standard (Figure S3). Using the same method, 16 pyrazines were studied.

In this study, a rapid direct injection method combined with UPLC–MS/MS for the determination of 16 pyrazines in Baijiu was developed. The MRM transitions, retention times, cone voltages and collision energies of the 16 pyrazines are summarized in Table 2. All pyrazine standards were used for identifying the retention times. The total ion chromatograms of pyrazines are presented in Figure S4. The obtained calibration curves were found to have good linearity, with a determination coefficient (*R*^2^) ≥ 0.99; the recoveries varied from 84.36% to 103.92%, and RSDs in triplicate of the samples were ≤6.36% (Table S3), indicating acceptable precision of the quantitative method.

### 3.2. Quantitation of Pyrazines and OAV Analysis

A total of 16 pyrazines were quantitated by UPLC–MS/MS. In all samples, the quantitative results (Table 3) showed that 2,3,5,6-tetramethylpyrazine (2356TTM, 475–1862 μg·L^−1^), 2,6-dimethylpyrazine (26DM, 460–1590 μg·L^−1^) and 2,3,5-trimethylpyrazine (235TM, 317–1755 μg·L^−1^) were the three most concentrated pyrazines. Lower concentrations were observed for 5-ethyl-2,3-dimethylpyrazine (5E35DM, 0.8–12.5 μg·L^−1^), 2-isobutyl-3-methylpyrazine (2I3M, 1.0–5.9 μg·L^−1^) and 2,3-diethyl-5-methylpyrazine (23DE5M, 1.1–15.5 μg·L^−1^). Using carbohydrates and amino acids/peptides as substrates, different model structures have been proposed to study the chemical synthesis of pyrazines in heat-treated foods [30]. In addition, pyrazines are biosynthesized by a few species of bacteria and fungi [31,32].

The OAVs were calculated to determine the contribution of pyrazines to the overall aroma of the SSAB (Table 3). The OAV estimation implied that four pyrazines contributed to the Baijiu’s overall aroma profile, since their concentrations surpassed odor thresholds. According to previous reports, and confirmed by this study, the important pyrazines for the aroma of Baijiu are 2-ethyl-3,5-dimethylpyrazine, 2-ethyl-6-methylpyrazine, 2,3,5-trimethylpyrazine and 2,6-dimethylpyrazine [3,33,34]. The highest OAV pyrazine in SSAB was 2-ethyl-3,5-dimethylpyrazine (2E35DM; OAVs: 11.2–69.5), followed by 2-ethyl-6-methylpyrazine (2E6M; OAVs: 6.4–17.3). This result is similar to previous research on roasted sesame-like flavor type Baijiu [33]. The OAVs of 2,6-dimethylpyrazine (OAVs: 0.6–2.0) and 2,3,5-trimethylpyrazine (OAVs: 0.4–2.4) were approximately 1. The OAV of 2,3-diethylpyrazine (23DE) ranged from 0.1 to 1.0 in in most SSAB samples. In addition, 2,3-dimethylpyrazine, 2,3-diethyl-5-methylpyrazine and 2-acetyl-3-methylpyrazine (2A3M) had OAVs ranging from 0.1 to 1.0 in all SSAB samples.

### 3.3. Relationships between Pyrazine Concentrations and Roasted Aroma.

Aroma compounds have similar structures or aroma properties and may create a superimposing effect when combined [20,21,22]. Pyrazine is a class of nitrogen-containing heterocyclic compounds, most of which have roasted aroma characteristics [35,36]. Some studies have shown that certain compounds can play a role in the overall aroma at levels below their odor thresholds [11,13,36]. This means that OAVs <1.0 may not necessarily indicate that the aroma compounds will not be perceived in the Baijiu. Therefore, in the current study, we were interested in whether pyrazines at sub-threshold concentrations contribute to the roasted aroma of SSAB by sensory interactions.

**Figure 1:**
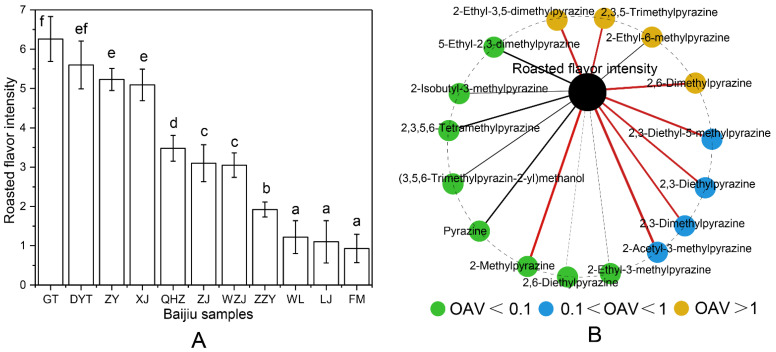
Roasted aroma intensities of the SSAB samples. The different letters indicate significant differences at α = 0.05 (**A**). Relationships between pyrazines and roasted aroma (**B**). Red line indicates a significant (*p* < 0.05) and strong (Spearman’s ρ > 0.5) correlation. The thickness of each connection (edge) between two nodes is proportional to Spearman’s correlation coefficient (ρ).

To analyze whether sub-threshold concentrations of pyrazines may contribute to the roasted aroma in SSAB, the correlation between pyrazine concentrations and roasted aroma intensity was analyzed by combining chemical and sensory analyses. First, the roasted aroma intensities of all of the SSAB samples were sensorially evaluated. The PanelCheck software (Nofima Mat, Longyearbyen, Norway) was used to analyze panelists (Figure S1). The Tucker-1 plot highlights roasted aroma attribute used in the profiling. The panel showed very good agreement as they were well clustered at the outer ellipse. The low MSE value suggested that panelists had good discrimination capacity. At the significance level of 5%, all *F* values surpassed the horizontal axis, which meant that the panelists had a good repeatability capability [26,27]. The SSAB samples came from different regions and brands, and the roasted aroma intensities ranged from 0.93 to 6.26 (Figure 1A). Then, we explored the correlations between pyrazine concentrations (Table 3) and roasted aroma intensities (Figure 1A) based on Spearman’s rank correlations (ρ > 0.5, *p* < 0.05) [24,29]. The results showed that eight pyrazines significantly correlated with roasted aroma, indicating that these eight pyrazines may be important contributors to the roasted aroma differences among the SSAB samples (Figure 1B).

Among the eight correlated pyrazines, 2-ethyl-3,5-dimethylpyrazine had a higher Spearman’s rank correlation, suggesting that it has a greater contribution to the roasted aroma. This was consistent with the observation that 2-ethyl-3,5-dimethylpyrazine had the highest OAVs (11.2–69.5) in the SSAB samples (Table 3). In addition, it is worth noting that the OAVs of five pyrazines were sub-threshold concentrations in most SSAB samples. Among them, the OAV of 2-methylpyrazine was much less than 0.1, which was less likely to have an effect on overall aroma, so it was not considered from the perspective of sensory interaction. Moreover, 2,3-Dimethylpyrazine, 2,3-diethylpyrazine, 2,3-diethyl-5-methylpyrazine and 2-acetyl-3-methylpyrazine were within the OAV range of 0.1 to 1.0. We were interested in whether the four pyrazines could contribute to the roasted aroma of SSAB.

### 3.4. Organoleptic Impact of Pyrazines on SSAB Aroma Perception

Studies have shown that some sub-threshold aroma compounds may contribute to the overall aroma by an additive effect with supra-threshold aroma compounds [11,21]. To analyze whether sub-threshold pyrazines can affect the supra-threshold pyrazines, which in turn have an impact on the roasted aroma in SSAB, four sub-threshold pyrazines and four supra-threshold pyrazines were selected for additional tests. Based on the quantitative and roasted aroma intensity results (Table 3 and Figure 1A), the pyrazines present at higher concentrations in the GT sample were added in corresponding amounts to the FM sample (the lowest roasted aroma intensity), using different experimental schemes (Table 4). Thus, the solution prepared in Table 4 was compared to the FM sample by 3-AFC tests. Based on the responses to the tests, the outcomes were evaluated statistically [37].

The first set of triangulation tests (Table 4; tests 1 to 8) involved assessing the individual perception of each pyrazine. There were no significant differences among 2,3-dimethylpyrazine, 2-acetyl-3-methylpyrazine, 2,3-diethylpyrazine and 2,3-diethyl-5-methylpyrazine (Table 4; tests 1 to 4). These results correspond to those of the odor thresholds in Table 3. It is worth noting that the four pyrazines added surpassed the odor thresholds in ethanol–water solution, but they nonetheless resulted in no significant difference in perception (Table 4; tests 5 to 8). No previous studies have stated the odor thresholds of these pyrazines in the Baijiu matrix; the odor thresholds were obtained from ethanol solution. These results demonstrated that the four supra-threshold pyrazines alone had no direct odor effects on SSAB, which may be because Baijiu is extremely rich in aroma compounds and is far more complex than the ethanol–water solution. However, the addition of four supra-threshold pyrazines combined was significantly perceived (*p* = 0.01) (Table 4; tests 9). In addition, when four sub-threshold pyrazines were added, more sensory panelists perceived a change in roasted aroma, and the significance level was reduced from 0.01 to 0.001 (Table 4; tests 10). The sensory analysis also revealed that the roasted aroma intensity of FM was significantly increased (Figure S5). The results show that the sub-threshold pyrazines affect the overall sensory properties of SSAB. In combination, 2,3-dimethylpyrazine, 2,3-diethylpyrazine, 2,3-diethyl-5-methylpyrazine and 2-acetyl-3-methylpyrazine impacted the roasted aroma at concentrations of approximately 19%, 46%, 67% and 39% of their odor thresholds, respectively. This is the first time it has been reported that mixing sub-threshold pyrazines could contribute to the overall aroma in Baijiu.

### 3.5. Olfactory Properties of Pyrazines Present at Sub-Threshold Concentrations

**Figure 2:**
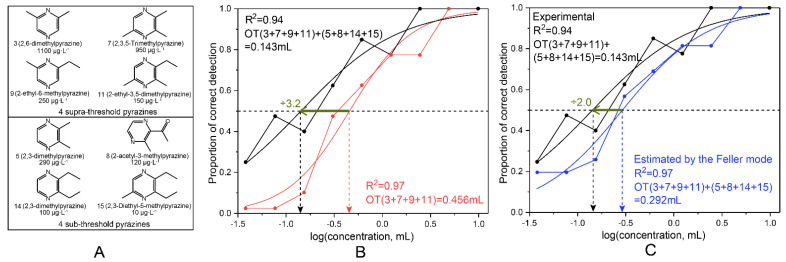
Four supra-threshold pyrazines and four sub-threshold pyrazines in most SSAB samples (number corresponds to Table 2) (**A**). Effect of four sub-threshold pyrazine addition on the detection probability of four supra-threshold pyrazines (**B**). Detection probability of four supra-threshold pyrazines determined experimentally and calculated according to Feller’s additive model (**C**). OT, odor threshold.

According to the roasted aroma intensity of SSAB samples, the QHZ sample had an intermediate-intensity roasted aroma. Thus, the concentrations of eight pyrazines in QHZ were selected for the next study (Figure 2A). Comparing the odor thresholds of four supra-threshold pyrazines in different matrices between ethanol–water solution and four sub-threshold pyrazines ethanol–water solution, the odor threshold was 0.456 mL in ethanol–water solution and 0.143 mL in four sub-threshold pyrazines ethanol–water solution; the odor threshold was decreased by a factor of 3.2 (from 0.456 to 0.143 mL) (Figure 2B). The addition of four sub-threshold pyrazines had significant effects on the odor threshold of four supra-threshold pyrazines. These findings confirm that through perceptual interactions, pyrazines at sub-threshold concentrations function as roasted aroma enhancers.

The researchers separated the relationships between aroma compounds into four groups [38]. To verify whether the sensory contribution of these compounds was due to a simple addition phenomenon or excessive addition effect, the influence of the inclusion of four sub-threshold pyrazines on the odor of the four supra-threshold pyrazines was analyzed. The sensory test results showed that the probability of experimental detection of the roasted aroma in the blend was greater than the calculated value (Table S4). The estimated roasted aroma odor threshold was 0.143 mL, 2.0 times less than the calculated odor threshold (0.292 mL) (Figure 2C), revealing an excessive addition effect after the addition of sub-threshold pyrazines to the supra-threshold pyrazines.

## 4. Conclusions

In this work, a total of 16 pyrazines were analyzed using UPLC–MS/MS in SSAB. Moreover, the sensory interactions of pyrazines were studied for the first time. The Spearman’s rank correlations indicated some sub-threshold pyrazines that were correlated with a roasted aroma, based on their concentrations, and roasted aroma intensity in SSAB samples. These findings showed the indirect effect of 2,3-dimethylpyrazine, 2,3-diethylpyrazine, 2,3-diethyl-5-methylpyrazine and 2-acetyl-3-methylpyrazine, present at sub-threshold concentrations, on roasted aroma expression. The results highlighted the contribution of sub-threshold pyrazines to the aroma of Baijiu, which play a role in perceptual interactions as roasted aroma enhancers. These findings provide new insight into the role of pyrazines in food aroma. This strategy can be used to recognize other significant aroma compounds in sub-threshold concentrations that can influence food aroma.

## Figures and Tables

**Table 1:** Composition of pyrazines to odor threshold determination in various matrices.

Reconstitution	Pyrazines Reconstitution Diluted in 15 mL of Matrix (mL)	Matrix
26DM + 2E6M + 235TM + 2E35DM	0.039/0.077/0.154/0.307/ 0.614/1.228/2.456/4.912/9.824	53% ethanol/water solution
		23DM + 23DE + 23DE5M + 2A3M in 53% ethanol/water solution

26DM, 1110 μg·L^−1^; 2E6M, 250 μg·L^−1^; 235TM, 950 μg·L^−1^; 2E35DM, 150 μg·L^−1^; 23DM, 290 μg·L^−1^; 23DE, 100 μg·L^−1^; 23DE5M, 11 μg·L^−1^; 2A3M, 120 μg·L^−1^.

**Table 2:** UPLC–MS/MS parameters for the pyrazines in soy sauce aroma type Baijiu (SSAB).

No.	Compounds	Retention Time (min) ± Standard Deviation	Precursor Ion (*m*/*z*)	Quantification Ion (*m*/*z*)	Cone Voltage (V)	Collision Energy (eV)	Confirmation Ion (*m*/*z*)	Cone Voltage (V)	Collision Energy (eV)
1	Pyrazine	1.77 ± 0.03	80.7	54.1	35	14	41.2	37	16
2	2M	3.35 ± 0.03	94.7	68.0	33	14	54.3	33	16
3	26DM	6.45 ± 0.05	108.9	41.5	31	19	67.7	33	18
4	TM2YM	7.05 ± 0.04	152.7	53.2	7	22	94.1	9	22
5	23DM	7.41 ± 0.06	108.9	41.5	31	19	67.7	33	18
6	2356TTM	8.41 ± 0.04	137.0	55.3	31	21	42.1	31	24
7	235TM	9.75 ± 0.03	123.0	82.2	33	17	42.0	33	20
8	2A3M	14.00 ± 0.07	136.8	109.1	17	16	94.5	17	18
9	2E6M	15.43 ± 0.06	123.0	108.2	27	19	81.5	29	19
10	2E3M	16.29 ± 0.08	123.0	108.2	27	19	81.5	29	19
11	2E35DM	18.76 ± 0.04	137.0	121.4	29	17	80.4	30	19
12	5E35DM	19.49 ± 0.06	137.0	121.4	29	17	80.4	30	19
13	26DE	25.48 ± 0.06	136.8	121.8	33	20	108.4	33	22
14	23DE	26.26 ± 0.09	136.8	121.8	33	20	108.4	33	22
15	23DE5M	29.43 ± 0.04	150.8	136.0	35	16	122.5	35	18
16	2I3M	32.64 ± 0.03	151.0	108.0	29	26	135.8	28	23
17	2-Propylpyrazine (IS)	20.73 ± 0.05	122.7	108.0	23	16	61.1	23	18

2M, 2-methylpyrazine; 26DM, 2,6-dimethylpyrazine; TM2YM, (3,5,6-trimethylpyrazin-2-yl)methanol; 23DM, 2,3-dimethylpyrazine; 2356TTM, 2,3,5,6-tetramethylpyrazine; 235TM, 2,3,5-trimethylpyrazine; 2A3M, 2-acetyl-3-methylpyrazine; 2E6M, 2-ethyl-6-methylpyrazine; 2E3M, 2-ethyl-3-methylpyrazine; 2E35DM, 2-ethyl-3,5-dimethylpyrazine; 5E35DM, 5-ethyl-2,3-dimethylpyrazine; 26DE, 2,6-diethylpyrazine; 23DE, 2,3-dimethylpyrazine; 23DE5M, 2,3-diethyl-5-methylpyrazine; 2I3M, 2-isobutyl-3-methylpyrazine.

**Table 3:** Odor thresholds, quantitative data (mean value of triplicate samples ± relative standard deviation), odor activity values (OAVs) and concentrations (mean value of all samples) in SSAB samples.

No.	Compounds	Threshold (μg·L^−1^)	GT ^d^	WZJ	ZY	QHZ	DYT	XJ	LJ	ZJ	WL	FM	ZZY	Mean Value of All Samples
			Concentration (μg·L^−1^)											
1	Pyrazine ^a^	300,000 ^b^	82.11 ± 4.08	192.41 ± 2.1	85.25 ± 3.66	176.21 ± 5.02	196.79 ± 1.45	193.92 ± 1.06	71.14 ± 0.67	117.55 ± 2.23	99.8 ± 4.11	73.08 ± 3.8	29.81 ± 4.5	119.82
2	2M ^a^	30,000 ^b^	135.21 ± 1.17	110.54 ± 3.64	148.2 ± 0.65	195.29 ± 0.59	159.41 ± 1.97	115.33 ± 2.2	62.15 ± 5.41	36.02 ± 3.41	71.56 ± 3.09	56.88 ± 2.16	51.06 ± 1.97	103.79
3	26DM	791 [3]	1257.48 ± 3.24 (1.6)	992.28 ± 1.97 (1.3)	1497.57 ± 3.96 (1.9)	1116.28 ± 6.09 (1.4)	1054.61 ± 0.88 (1.3)	951.06 ± 1.28 (1.2)	735.28 ± 0.69 (0.9)	1589.16 ± 0.47 (2.0)	878.3 ± 2.78 (1.1)	459.48 ± 4.6 (0.6)	618.19 ± 1.06 (0.8)	1013.61
4	TM2YM ^a^	267,946 ^c^	6.51 ± 1.76	74.7 ± 2.89	53.07 ± 3.37	54.1 ± 1.92	25.4 ± 6.17	39.28 ± 3.06	7.05 ± 2.71	17.33 ± 4.32	10.54 ± 3.66	18.14 ± 2.79	10.92 ± 1.78	28.82
5	23DM	965 ^c^	312.01 ± 3.96 (0.3)	175.8 ± 2.24 (0.2)	682.51 ± 2.09 (0.7)	290.8 ± 1.88 (0.3)	195.26 ± 3.62 (0.2)	315.7 ± 3.84 (0.3)	112.66 ± 0.77 (0.1)	295.3 ± 2.6 (0.3)	237.4 ± 4.78 (0.2)	125.89 ± 3.5 (0.1)	167.2 ± 0.88 (0.2)	264.59
6	2356TTM ^a^	80,100 [3]	1861.98 ± 7.09	1634.52 ± 1.02	805.19 ± 0.56	1208.3 ± 1.70	895.22 ± 0.79	1183.12 ± 4.41	474.99 ± 4.27	1609.47 ± 2.28	601.51 ± 4.04	693.42 ± 2.19	886.07 ± 4.78	1077.62
7	235TM	730 [3]	1086.25 ± 0.71 (1.5)	1328.06 ± 1.37 (1.8)	866.33 ± 1.05 (1.2)	950.19 ± 1.36 (1.3)	760.57 ± 5.81 (1.0)	906.55 ± 5.52 (1.2)	440.1 ± 0.83 (0.6)	1754.48 ± 1.27 (2.4)	497.11 ± 3.74 (0.7)	316.52 ± 1.53 (0.4)	513.97 ± 4.24 (0.7)	856.38
8	2A3M	415 ^c^	236.75 ± 3.5 (0.6)	156.34 ± 0.57 (0.4)	79.26 ± 2.97 (0.2)	125.41 ± 4.43 (0.3)	196.22 ± 5.09 (0.5)	261.07 ± 1.76 (0.6)	78.15 ± 1.69 (0.2)	56.12 ± 3.56 (0.1)	65.4 ± 1.86 (0.2)	72.3 ± 3.71 (0.2)	49.49 ± 3.55 (0.1)	125.13
9	2E6M	40 [33]	428.24 ± 2.04 (10.7)	330.72 ± 1.45 (8.3)	502.11 ± 0.54 (12.6)	255.9 ± 4.15 (6.4)	420.85 ± 6.20 (10.5)	369.26 ± 0.63 (9.2)	361.98 ± 6.02 (9.0)	690.69 ± 0.57 (17.3)	400.24 ± 7.64 (10.0)	336.76 ± 2.3 (8.4)	293.25 ± 5.29 (7.3)	399.10
10	2E3M ^a^	297 ^c^	8.19 ± 0.74	19.33 ± 0.62	6.49 ± 2.51	23.1 ± 4.28	19.84 ± 4.2	7.11 ± 3.79	6.19 ± 2.09	46.19 ± 4.92	5.1 ± 0.79	6.71 ± 0.33	2.65 ± 2.81	13.72
11	2E35DM	7.5 [33]	389.85 ± 2.04 (52.0)	294.45 ± 2.55 (39.3)	240.44 ± 0.76 (32.1)	153.61 ± 1.68 (20.5)	486.49 ± 3.96 (64.9)	251.42 ± 4.65 (33.5)	100.23 ± 0.77 (13.4)	521.57 ± 2.14 (69.5)	112.3 ± 3.01 (15.0)	83.75 ± 4.18 (11.2)	95.1 ± 4.21 (12.7)	248.11
12	5E23DM ^a^	530 ^b^	1.83 ± 4.22	6.42 ± 1.09	1.64 ± 0.41	2.04 ± 1.56	5.68 ± 2.8	12.52 ± 4.55	1.01 ± 3.09	1.64 ± 2.47	0.83 ± 0.85	1.75 ± 2.76	1.23 ± 3.09	3.33
13	26DE	296 ^c^	25.33 ± 2.19 (<0.1)	39.57 ± 3.44 (0.1)	31.09 ± 1.09 (0.1)	11.75 ± 0.77 (<0.1)	43.21 ± 2.59 (0.1)	52.41 ± 6.78 (0.2)	75.21 ± 4.58 (0.3)	17.08 ± 3.92 (<0.1)	12 ± 2.06 (<0.1)	17.63 ± 3.9 (<0.1)	15.33 ± 2.77 (<0.1)	30.96
14	23DE	172 [3]	159.15 ± 3.9 (0.9)	240.14 ± 4.83 (1.4)	125.61± 5.24 (0.7)	107.55 ± 2.88 (0.6)	167.61 ± 1.89 (1.0)	110.9 ± 3.06 (0.6)	64.8 ± 4.71 (0.4)	97.14 ± 2.09 (0.6)	62.4 ± 5.11 (0.4)	78.6 ± 4.3 (0.5)	28.08 ± 3.11 (0.2)	112.91
15	23DE5M	18 ^c^	13.76 ± 2.81 (0.8)	10.54 ± 1.97 (0.6)	15.47 ± 3.35 (0.9)	11.86 ± 6.09 (0.7)	4.5 ± 1.44 (0.3)	6.69 ± 3.79 (0.4)	4.39 ± 4.29 (0.2)	2.71 ± 3.09 (0.2)	4.16 ± 6.5 (0.2)	1.7 ± 3.72 (0.1)	1.09 ± 1.03 (0.1)	6.99
16	2I3M ^a^	130 ^b^	2.31 ± 5.09	5.87 ± 2.78	4.25 ± 1.83	4.25 ± 1.09	1.33 ± 4.62	1.41 ± 3.7	3.82 ± 2.88	0.96 ± 6.47	1.14 ± 4.55	1.92 ± 3.29	3.18 ± 2.74	2.77

^a^ The compounds with OAVs <0.1 were calculated in all SSAB samples in this study. ^b^ Odor thresholds were taken from www.thresholdcompilation.com (accessed on 6 November 2020). ^c^ Odor thresholds in 53% ethanol/water solution (*v*/*v*) detected in this study. ^d^ The SSAB samples were selected for this study. The detailed information is given in Table S1.

**Table 4:** Olfactory impact of the addition of various pyrazines in SSAB sample (FM).

Compounds	23DM	2A3M	23DE	23DE5M	26DM	235TM	2E6M	2E35DM	Difference Observed
Concn (μg·L^−1^)	180	160	80	12	800	770	90	300	
OAV	0.19	0.39	0.46	0.67	1.00	1.10	2.25	40.00	
test 1	√	×	×	×	×	×	×	×	ns
test 2	×	√	×	×	×	×	×	×	ns
test 3	×	×	√	×	×	×	×	×	ns
test 4	×	×	×	√	×	×	×	×	ns
test 5	×	×	×	×	√	×	×	×	ns
test 6	×	×	×	×	×	√	×	×	ns
test 7	×	×	×	×	×	×	√	×	ns
test 8	×	×	×	×	×	×	×	√	ns
test 9	×	×	×	×	√	√	√	√	**
test 10	√	√	√	√	√	√	√	√	***

***, 0.1% significant level; **, 1% significant level; ns, *p* ≥ 0.05 (not significant); √, presence of listed compounds; and ×, absence of listed compound.

## Data Availability

The data presented in this study are available on request from the corresponding author.

## Supplementary Materials

The following are available online at https://www.mdpi.com/2304-8158/10/2/441/s1, Figure S1: Tucker-1 (A) plots visualizing the repeatability; MSE plot (B) visualizing the repeatability; F plots (C) visualizing the panels’ ability to discriminate between the SSAB samples for roasted attribute, Figure S2: MS/MS method optimization (A and B) and mass spectra (C) for 2,3-diethyl-5-methylpyrazine; Figure S3: Comparison of the chromatograms of an authentic standard (A) of 2,3-diethyl-5-methylpyrazine and its peak in the sample (B), Figure S4: Total ion chromatograms all pyrazine under the optimal instrumental conditions, Figure S5: Roasted aroma intensities of the FM, Table S1: Information of soy sauce aroma type Baijiu samples in this study, Table S2: The complementary nominal scale for sensory analysis, Table S3: Linear range, *R*^2^, LOD, and LOQ of the established method for pyrazines, Table S4: Determination of the detection probability (*p*) of mixture of four supra-threshold pyrazines and four sub-threshold pyrazines: measured aroma threshold and calculated aroma threshold obtained by Feller’s additive model.
