# Supplementary material for: Quantitative Analysis of Pyrazines and Their Perceptual Interactions in Soy Sauce Aroma Type Baijiu
Source: Foods. 2021 Feb 17;10(2):441. doi: 10.3390/foods10020441 (PMC7922815; doi:10.3390/foods10020441)
Supplement: Supplementary file 1 [file foods-10-00441-s001.pdf]

Supplementary Material

Quantitative Analysis of Pyrazines and Their Perceptual Interactions in Soy Sauce Aroma Type Baijiu

Yan Yan [1,2], Shuang Chen [1], Yao Nie [1,*], and Yan Xu [1,*]

[1]State Key Laboratory of Food Science & Technology, Key Laboratory of Industrial Biotechnology of Ministry of Education & School of Biotechnology, Jiangnan University, Wuxi 214100, China;

[2]School of Liquor and Food Engineering, Guizhou University, Guiyang 550025, China.

*Correspondence: ynie@jiangnan.edu.cn (Y.N.); yxu@jiangnan.edu.cn (Y.X.).

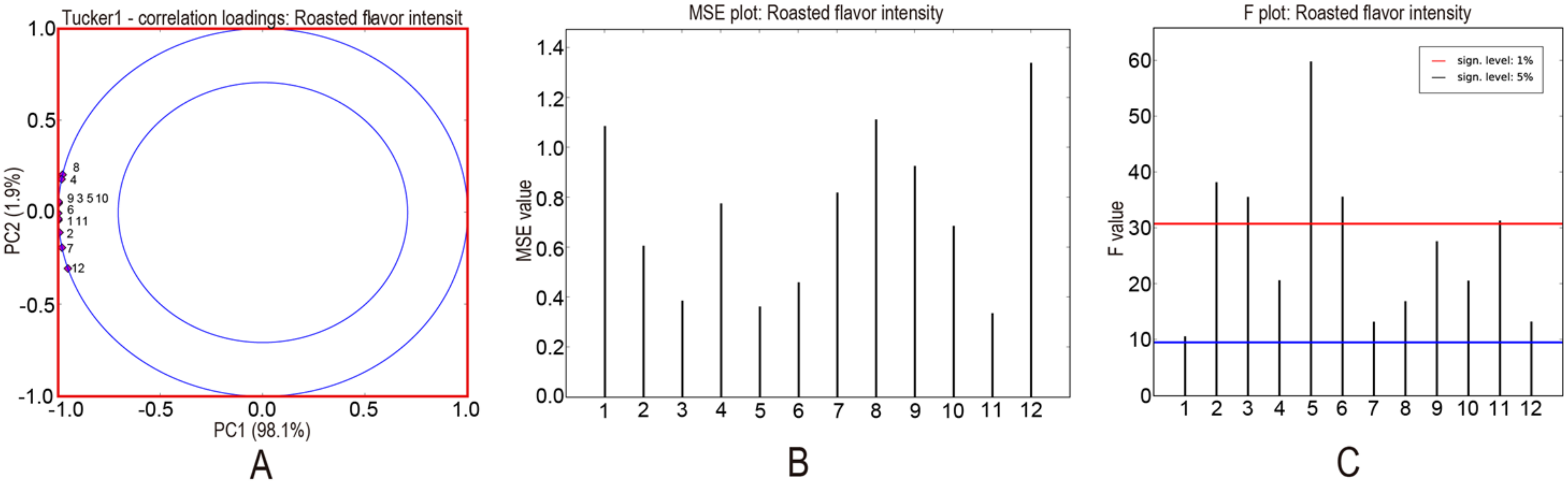

Supplemental Figure S1: Tucker-1 (A) plots visualizing the agreement. MSE plot (B) visualizing the repeatability. *F* plots (C) visualizing the panels' ability to discrimination between the Baijiu samples for roasted attribute.

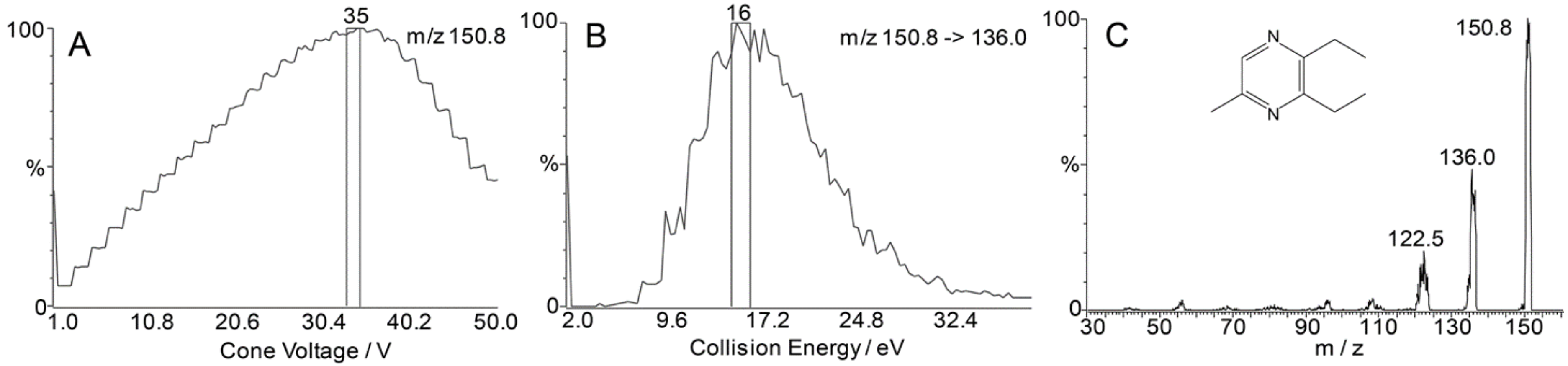

Supplemental Figure S2: MS/MS method optimization (A and B) and mass spectra (C) for 2,3-diethyl-5-methylpyrazine.

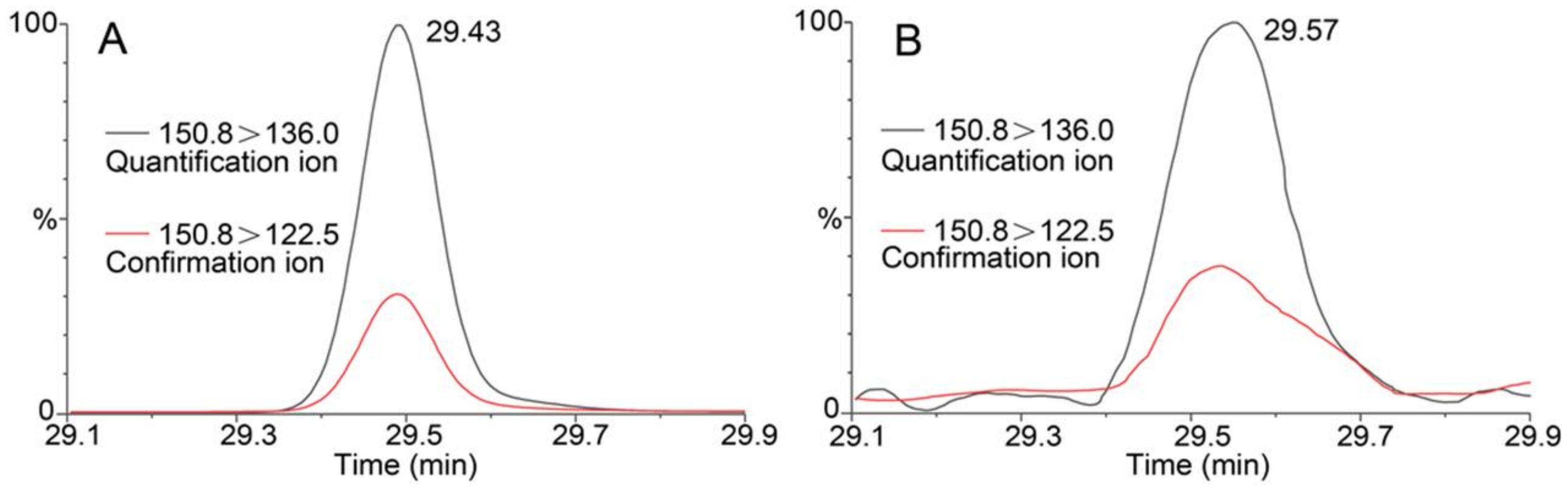

Supplemental Figure S3: MS/MS method optimization (A and B) and mass spectra (C) for 2,3-diethyl-5-methylpyrazine.

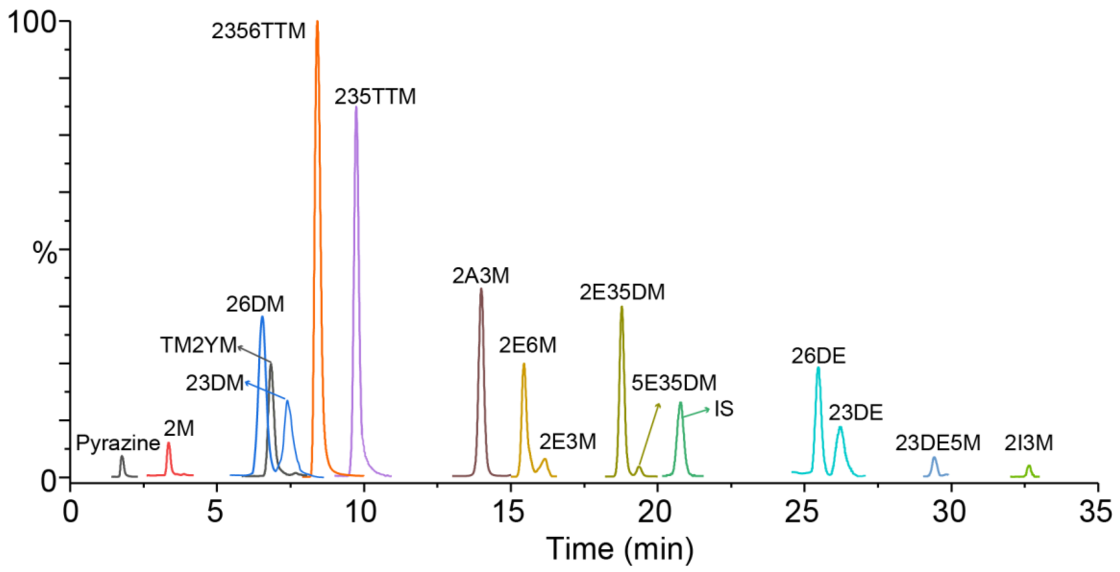

Supplemental Figure S4: Total ion chromatograms all pyrazine standards under the optimal instrumental conditions.

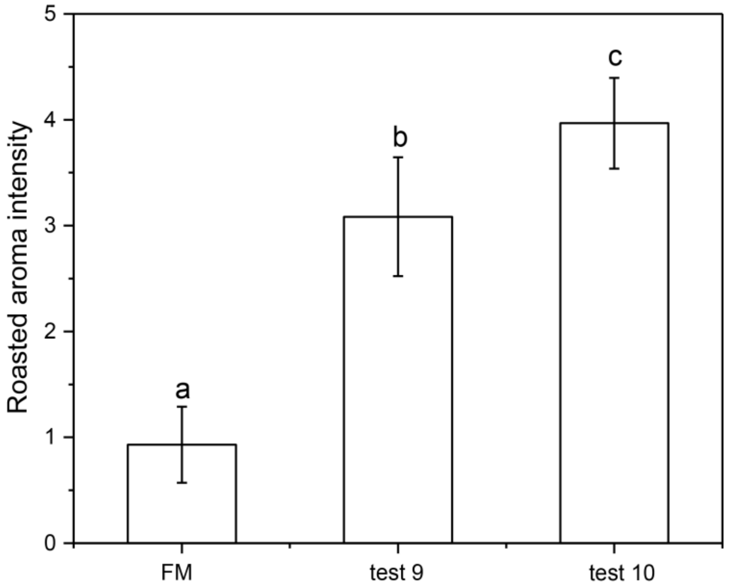

Supplemental Figure S5: Roasted aroma intensities of the FM.

Table S1. Information of soy sauce aroma type Baijiu samples in this study

No.	Name	Abbreviation	Manufacturer	Location	Alcohol % by Volume
1	GUO TAI GUO BIAO	GT	GUI ZHOU GUOTAI LIQUOR CO., LTD.	Beijing, China	53
2	MOUTAI PRINCE	WZJ	KWEICHOW MOUNTAI CO., LTD.	Zunyi, China	53
3	ZHAI YAO	ZY	GUI ZHOU JINSHAJIAO LIQUOR CO., LTD.	Zunyi, China	53
4	QIN HAN ZHANG JIU	QHZ	KWEICHOW MOUNTAI QINHANZHANG JIUYE CO., LTD.	Zunyi, China	53
5	DIAOYUTAI AMBASSADOR LIQUOR	DYT	KWEICHOW DIAOYUTAI STATE GUEST DISTILLERY CO., LTD.	Zunyi, China	53
6	JIAO CANG	XJ	GUIZHOU MAOTAI OISTILLERY(GROUP) XI JIU CO., LTD.	Zunyi, China	53
7	LANG PAI LANG JIU	LJ	SICHUAN GULIN LANGJIU DISTILLERY CO., LTD.	Luzhou, China	53
8	ZHEN WU	ZJ	GUIZHOU ZHENJIU LIQUOR-MAKING CO., LTD.	Zunyi, China	53
9	WU LING JIU	WL	CHINA HUNAN WULING SPIRITS CO., LTD.	Changde, China	53
10	FU MAO	FM	FU MAO WINE GROUP CO., LTD.	Fujian, China	50
11	ZHEN ZHU YE	ZZY	HU BEI ZHEN ZHU YE LIQUOR CO., LTD.	Xiangyang, China	53

Table S2. The complementary nominal scale for sensory analysis.

Intensity	Rating range
The attribute is not perceived at all	0
Doubts about the presence of the attribute	0-1
The attribute is perceived but very slightly	1-2
The attribute is clearly perceived, although it is slight	2-3
The attribute is clearly perceived, but the intensity is lower than the reference	3-4
The attribute is clearly perceived, and the intensity is very close to the reference	4-5
The intensity of the attribute is similar to the reference	5
The intensity of the attribute is slightly higher than the reference	5-6
The intensity of the attribute is much higher than the reference	6-7

Table S3. Linear range, R^2, LOD, and LOQ of the established method for pyrazines.

No.	Compounds	Linear equation (μg·L⁻¹)	Linear range (μg·L⁻¹)	R^2	LOD[a] (μg·L⁻¹)	LOQ[b] (μg·L⁻¹)	RSD[c] (%)	Recovery (%)
1	Pyrazine	y = 0.0093x + 8.561	7-896	0.9991	1.92	6.35	4.33	89.01
2	2M[a]	y = 0.0019x - 1.249	10-1280	0.9975	1.86	6.15	2.90	94.77
3	26DM	y = 0.0275x + 18.568	5-5120	0.9970	1.24	4.09	3.96	84.36
4	TM2YM	y = 0.0011x + 0.0032	5-1280	0.9985	0.53	1.77	2.10	87.20
5	23DM	y = 0.0685x - 22.106	10-1280	0.9995	1.03	3.41	6.36	89.62
6	2356TTM	y = 0.0014x - 64.603	5-5120	0.9918	0.63	2.08	3.01	97.29
7	235TM	y = 0.0024x + 34.802	13-6656	0.9982	0.91	3.01	1.22	92.08
8	2A3M	y = 0.0189x + 55.21	6-768	0.9969	1.43	5.06	3.59	85.61
9	2E6M	y = 0.0029x + 28.814	2-4096	0.9996	0.76	2.53	1.86	95.07
10	2E3M	y = 0.0068x +7.034	2-512	0.9979	0.41	1.35	4.16	86.10
11	2E35DM	y = 0.0004x + 0.0066	2-4096	0.9997	0.56	1.84	1.02	86.22
12	5E35DM	y = 0.0059x + 0.0043	2-512	0.9975	0.33	1.10	5.10	87.42
13	26DE	y = 0.018x - 24.705	2-512	0.9992	0.28	0.95	1.76	95.07
14	23DE	y = 0.0275x - 49.33	5-1280	0.9986	0.45	1.50	1.09	84.59
15	23DE5M	y = 0.0023x + 0.0124	0.7-89.6	0.9986	0.15	0.51	6.17	95.19
16	2I3M	y = 0.0011x + 0.0032	0.7-89.6	0.9991	0.17	0.58	2.38	103.92

[a]LOD, limit of detection; [b]LOQ, LOQ, limit of quantitation; [c]RSD, relative standard deviation.

Table S4. Determination of the detection probability (*p*) of mixture of four sub-threshold pyrazines (A) and supra-threshold pyrazines (B): measured aroma threshold and calculated aroma threshold obtained by Feller's additive model.

before mixture		after mixture	
p(A) alone	*p*(B) alone	*p*(B in "A" sample) experimental	*p*(B in "A" sample) calculated according to Feller's additive model
calculated and fixed	= ((3 × proportion of correct responses) − 1)/2		$= p(A) + p(B) - p(A)\,p(B)$
0.175	0.025	0.25	0.196
0.175	0.025	0.475	0.196
0.175	0.1	0.4	0.258
0.175	0.475	0.625	0.567
0.175	0.625	0.85	0.691
0.175	0.775	0.775	0.814
0.175	0.775	1	0.814
0.175	1	1	1
0.175	1	1	1

A, four sub-threshold pyrazines (2,3-dimethylpyrazine, 2,3-diethylpyrazine, 2,3-diethyl-5-methylpyrazine, and 2-acetyl-3-methylpyrazine); B, four supra-threshold pyrazines (2,6-dimethylpyrazine, 2-ethyl-6-methylpyrazine, 2,3,5-trimethylpyrazine, and 2-ethyl-3,5-dimethylpyrazine).
